# Mitochondrial DNA copy number is associated with incident chronic kidney disease and proteinuria in the AIDS linked to the intravenous experience cohort

**DOI:** 10.1038/s41598-023-45404-9

**Published:** 2023-10-27

**Authors:** Sakshi R. Tewari, Gregory D. Kirk, Dan E. Arking, Jacquie Astemborski, Charles Newcomb, Damani A. Piggott, Shruti Mehta, Gregory M. Lucas, Jing Sun

**Affiliations:** 1 Department of Epidemiology, Johns Hopkins University, Bloomberg School of Public Health, Baltimore, USA; 2 Department of Medicine, Johns Hopkins University, Baltimore, MD USA; 3 Department of Genetic Medicine, McKusick-Nathan Institute, Johns Hopkins University School of Medicine, Baltimore, MD USA

**Keywords:** Public health, Biomarkers, Medical research, Nephrology, Risk factors

## Abstract

We evaluated the prospective association of mitochondrial DNA copy number (mtDNA CN) with markers of kidney function among a cohort of persons who inject drugs (PWID). This is a Prospective cohort study nested in the AIDS linked to the intravenous experience cohort (community-based cohort of PWID in Baltimore, MD). mtDNA CN was measured at two time-points 5 years apart using a real-time polymerase chain reaction. Kidney function (estimated glomerular filtration rate [eGFR], serum creatinine, urine protein) was measured annually. We used linear mixed effects models to evaluate kidney function trajectories (N = 946) and Cox regression models to assess hazard of incident CKD (eGFR < 60 at two consecutive visits, N = 739) and proteinuria (urine protein:creatinine ratio > 200, N = 573) by level of mtDNA CN (Low [lowest quartile], vs high [other three quartiles]. Models were adjusted for demographic and behavioral characteristics, HIV and/or HCV infection, and comorbidity burden. Low mtDNA CN was independently associated with higher hazard of incident CKD (aHR: 2.33, 95% CI 1.42, 3.80) and proteinuria (aHR: 1.42, 95% CI 1.04, 1.96). Participants with low mtDNA CN had greater declines in eGFR and greater increases in serum creatinine over time. Low mtDNA CN is associated with more rapid kidney function decline and risk of incident CKD and proteinuria.

## Introduction

### Background and rationale

With a prevalence of 15% among US adults^1,2^, chronic kidney disease (CKD) is associated with increased cardiovascular events^3^, end stage renal disease^4,5^, hospitalization and mortality^6,7^. CKD refers to the sustained loss of kidney function and is classified based on cause^8^. Risk factors for CKD include hypertension, diabetes, smoking, and older age^9–11^. Kidney disease risk is pronounced in people with HIV (PWH) compared to those without; with 3–6 times higher proteinuria risk^12–15^. Other studies demonstrate that mean GFR can be 10–20 units lower among PLWH compared to negative controls^16,17^. This is attributable to both a high prevalence of classic CKD risk factors (i.e. diabetes, hypertension, etc.) and chronic inflammation due to HIV. Specifically, HIV viral products^18,19^ and some antiretroviral therapies (ART)^20^ may directly interfere with kidney and immune cell metabolic processes. In particular, long term HIV treatment with protease inhibitors and nucleotide reverse-transcriptase inhibitors is associated with mitochondrial dysfunction^21–25^. Additionally, nephrotoxic effects of ART drug cocktails, or individual drugs like Tenofovir, have been demonstrated by studies showing 1.3 times increased risk of proteinuria per year of Tenofovir exposure^26^, 1.3 and 3 times higher odds of > 3% annual eGFR decline among ART users vs HIV positive ART naïve and HIV negative participants respectively^15^, higher prevalence of CKD stages 3–5 after ART treatment^27^, and tubular proteinuria persistence to name a few^16,28^. Risk factors of illicit drug use^29,30^, and opportunistic infections like hepatitis C virus (HCV) coinfection^31^ also independently or jointly increase CKD risk.

Of all the key populations at highest risk of HIV^32^, persons who inject drugs (PWID) face a unique challenge due to exposure to high prevalence of HCV coinfection, polysubstance use, and polypharmacy (ART and comorbidities medications). The higher drug burden on kidneys, as well as adverse drug-drug interactions^33^ increase susceptibility for adverse kidney related outcomes compared to other PWH. A meta-analysis showed that sharing syringes and drug preparation materials is associated with a ~ 2 fold increase in risk of HCV seroconversion^34^, which can contribute to kidney function decline via accumulation of viral antigen–antibody complexes and viral effects^35,36^. This study also reported population attributable risk ranging between 25 and 43%, implying the percent by which HCV seroconversion can reduce upon elimination of these behaviors in the PWID population. The etiology of CKD among PWH and PWID, therefore, is multifactorial and often results from complex exposures to several risk factors.

Previous studies suggest that damage to kidney cell mitochondrial characteristics (DNA, protein etc.), may be related to poor kidney outcomes due to disturbances in mitochondrial membrane potential, reactive oxygen species (ROS) production or aerobic respiration^37–39^. Inversely, some studies have demonstrated that exposure to agents bolstering mitochondrial function in kidney cells enhances their recovery from injury^40,41^. Mitochondria are organelles that produce cellular energy in the form of Adenosine Tri Phosphate [ATP] via oxidative phosphorylation (OXPHOS), of which ROS are a by-product. Normally, ROS is maintained at low level by antioxidant pathways and act as secondary messengers^42^. However, loss of ROS balance is a characteristic of mitochondrial dysfunction, and markers of oxidative stress due to ROS are often elevated among PLWH compared to controls^43,44^ linked to HIV viral products and treatments^45^. The interplay of ROS imbalance and mitochondrial dysfunction can be detrimental to cell metabolism and cell repair. Mitochondrial DNA (mtDNA) content is a surrogate for mitochondrial function, because mitochondrial function is difficult to measure^46^. Its wide use in research is based on the idea that high ROS levels can imbalance the replisome machinery of mtDNA, thereby reducing its copy number (CN)^47–51^. mtDNA CN has been used as a surrogate for mitochondrial function in large-scale cohort studies examining a range of outcomes with the notable ARIC study (Tin et al.^57^) that demonstrated lower CKD incidence in highest mtDNA CN quartile compared to lowest (HR: 0.65, *P* < 0.001) over a 20-year median follow-up of older adults^52–58^.

Despite the scientific advances in kidney disease research over the last few decades, CKD remains a ‘silent killer’. Indeed, most patients (90%) are unaware they have CKD due to lack of clinical symptoms until reaching an advanced stage^1^, emphasizing the importance of subclinical CKD endpoints. Additionally, due to the long natural history of CKD, “hard” CKD endpoints (such as death or end-stage renal disease [ESRD]) have posed a research barrier with respect to lengthy follow-up requirements for population-level studies. These limitations created the need for easily measured surrogates that occur earlier in the CKD natural history and are specific to its process^59,60^. Well accepted CKD surrogates include percentage declines in Estimated Glomerular Filtration Rate (eGFR)^61,62^, as well as clinical cut-offs for eGFR and proteinuria^63,64^.

CKD detection efforts have focused on understanding CKD etiology at the genetic^65,66^ and molecular scales^67,68^ at the population level, which has implications in biomarker discovery for detection, risk stratification and therapeutics. This is crucial, as early detection and treatment could potentially impact long-term survival and outcomes among patients on the pathway to CKD^69,70^. Continued efforts to understand CKD etiology and to develop screening and monitoring tools represent a research priority.

While previous in vitro studies have shown mitochondrial dysfunction in kidney and peripheral blood cells is linked with poor kidney function^37,40,71–73^ there are limited population-level studies examining this longitudinal association. Indeed, the focus on the PWID key population is even more sparse, as are studies of how HIV status might affect mtDNA function. The main objective of this study is to investigate the longitudinal association between mtDNA CN and kidney function in a population of people with a history of injection drug use.

## Methods

**Figure 1:**
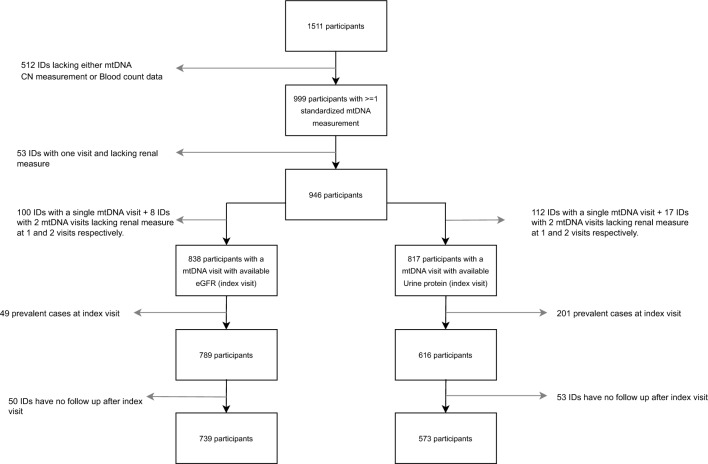
Sample selection flowchart for analyses.

The AIDS Linked to the Intravenous Experience (ALIVE) study is a community-based prospective cohort of current or previous PWID in Baltimore, MD. Since 1988, participants have attended follow-up visits semi-annually to answer structured interviews on health and behavior and undergo a physical examination and biospecimen collection^74^. Our data included ALIVE participants followed between 2005 and 2019 (Fig. 1). All participants provided informed consent and the study was approved by the Johns Hopkins IRB. All study methods were performed in accordance with the relevant guidelines and regulations. The current study followed the Strengthening the Reporting of Observational Studies in Epidemiology (STROBE) reporting guidelines.

### Measurements

#### Glomerular filtration rate, serum creatinine, urine protein, urine creatinine and incident CKD

Kidney function (serum creatinine concentration [reference range: 0.70–1.25 mg/dL], urine albumin, and urine creatinine) was ascertained at annual visits from 2005 to 2019. eGFR was estimated using the CKD-EPI equation^75^ from serum creatinine, age, sex and race data (reference range: > OR = 60 mL/min/1.73m^2^). We also calculated the race-neutral eGFR using the updated CKD-EPI (2021) formula for a supplementary analysis^76^. Missing kidney function measurements were imputed from the nearest visits within 1 year if available (of 7202 total visits in our mixed effects analysis; 7.6% eGFR and serum creatinine was missing and 3.6% imputed,10.8 urine protein was missing and 4.3% imputed, 10.2% creatinine was missing and 4.1% imputed). Prevalent CKD was defined as having baseline eGFR < 60 ml/min/1.732 m^2^ (considered stage 3 CKD). We defined incident CKD as eGFR < 60 at two consecutive visits in our main analysis. Prevalent proteinuria was defined as baseline urine protein to creatinine ratio (UPCR) > 200. Incident proteinuria was defined as the first occurrence of UPCR > 200. Additionally, we explored an additional outcome parameter reflecting kidney injury defined as the first occurrence of eGFR declining by at least 40% compared to baseline based on previous literatures^77–79^.

#### Mitochondrial DNA copy number (mtDNA CN) measurement

mtDNA CN was measured at 2 visits 5 years apart derived from the buffy coat layer isolated from peripheral blood samples by density centrifugation. DNA extraction was performed using the QIA symphony SP and the QIA symphony DSP DNA Midi Kit (Qiagen, Valencia, CA). After DNA was extracted, mtDNA CN was determined by a multiplexed real time quantitative polymerase chain reaction (qPCR) using ABI TaqMan chemistry (Applied Biosystems). The qPCR was carried out for amplification of two genes: mtDNA gene ND1 and nuclear DNA gene RPPH1, using separate assays. The assays were carried out on a 384 well plate in triplicate with each well carrying out a single assay in a total volume of 10 ul, out of which 20 ng was the DNA sample. Cycle threshold was used to identify the initial DNA concentration of the samples. For each participant, the cycle threshold for ND1 and RPPH1 genes was measured using the ABI Viia7 software and the difference calculated (ΔCt) to get the relative measure of mtDNA vs nuclear DNA. Measurements were adjusted for random effects of pipetting and plating using mixed effects models and then standardized by the standard deviation. mtDNA CN were then further standardized by cell composition of the samples (white blood cell [WBC] counts and platelet counts) using linear regression^80^. Cell compositions, especially WBC counts and platelet counts, could influence mtDNA CN in peripheral blood. This is because platelets do not contain nuclear DNA but do contain mtDNA, while white blood cell (WBC) counts were negatively correlated with mtDNA CNs (correlation coefficient, − 0.33).

#### Other covariates

Demographic characteristics such as age, sex, race/ethnicity and behavioral characteristics such as cigarette use (non-smoker, < 1 pack/day, ≥ 1 pack/day) and injection drug use (yes vs. no during last six months) were collected through standardized interviews. HIV serostatus was defined by detection of HIV-1 antibodies by an ELISA assay and confirmed by Western blot. HCV serostatus was defined by anti-HCV positivity and remained positive after the first positive results. Using blood pressure measurements at each visit, hypertension was defined as a systolic blood pressure ≥ 140 mmHg or a diastolic blood pressure ≥ 90 mmHg or self-reported use of anti-hypertensive medication. Diabetes was defined as HbA1c levels ≥ 6.5% or self-reported use of diabetic medication.

### Statistical methods

We compared baseline characteristics of participants across the mtDNA CN categories using *t* tests for normally distributed continuous variables (age), Wilcoxon tests for skewed continuous variables (BMI), and chi-squared tests for categorical variables. We conducted a survival analysis to evaluate the risk of incident CKD and proteinuria by level of mtDNA CN. Log-rank tests were performed and Kaplan–Meier curves were plotted by mtDNA CN categories. Multivariable Cox regression models were used to evaluate hazards for incident CKD and proteinuria by mtDNA CN groups, respectively. Models were controlled for demographic, behavioral and comorbidity burden. To further evaluate the association between mtDNA CN and trajectories of kidney function decline, we used linear mixed-effects model to evaluate the prospective association of eGFR, and separately, of log-transformed serum creatinine with mtDNA categories adjusted for baseline age, hypertension, diabetes, cigarette use, injection drug use, HIV status and HCV status. Confounders were selected based on existing literature, statistical significance in the univariate models, comparison of adjusted and unadjusted models, and data availability in the cohort. We controlled for race and sex in addition to other covariates in the serum creatinine models. Race and sex were included in the eGFR equation and were not controlled for in the mixed effects eGFR model, excluding which did not affect our estimates or their significance.

As there are no recommended clinical-cut offs for “high” versus “low” mtDNA CN, quartiles were created based on the distribution of mtDNA CN measurements from all measurements in the study population. The measurements were time updated and we assumed mtDNA CN remained constant between visits. During preliminary analyses, we evaluated the association of mtDNA CN with trajectories of kidney function, modeling mtDNA CN in 4 quartiles. We observed that the association between mtDNA CN and kidney function decline was not linear. Specifically, individuals in the lowest quartile of mtDNA CN had significantly faster decline in eGFR and serum creatinine compared to individuals in the higher 3 quartiles (Supp Fig. 1, Supp. Table 4). Additionally, our previous study^80^ demonstrated that lowest level of mtDNA CN had highest risk of adverse HIV outcomes while the other three quartiles shared similar risk. Therefore, we combined the higher 3 quartiles into one category (called higher mtDNA CN hereafter) and the lowest quartile as our exposed category (called low mtDNA CN hereafter) in the primary analyses, allowing us to focus on the risk phenotype. Analyses were performed using Stata version 16.1. Two-sided *P* values were used and statistical significance was set at < 0.05.

## Results

### Participant characteristics

Among 1511 active cohort participants between 2005 and 2019, 946 participants were included in the linear mixed effects analysis, while 739 participants were included in the survival analysis for incident CKD and 573 in survival analysis for proteinuria. The process of selecting analytic populations and the data availability for each analysis has been illustrated in Fig. 1. Among 946 participants with total 6900 person-years (median: 8 years, interquartile range [IQR]: 4–11) of follow-up, the median age at baseline was 49 years (IQR: 44–54), with the majority being black (87.2%), male (64.5%), smokers of less than 1 pack/day (57.7%) and HCV seropositive (85.3%). Those with low mtDNA CN were more likely to be actively injecting, HIV-positive, HCV seropositive and have lower BMI compared to those with higher mtDNA CN. (all *P* value < 0.05, Table 1).

**Table 1:** Participant characteristics at baseline by mitochondrial DNA copy number category.

Variable	Overall (N = 946)	Higher quartiles of mtDNA CN (N = 701)	Lowest quartile of mtDNA CN (N = 245)	*P* value*
Demographic factors				
Age, year, mean (SD)	48.6 (8.4)	48.6 (8.3)	48.6 (8.5)	**0.97**
Sex, % (n)				0.21
Male	64.5 (610)	63.3 (444)	67.8 (166)	
Female	35.5 (336)	36.7 (257)	32.2 (79)	
Race, % (n)				0.21
Black	87.2 (825)	88.0 (617)	84.9 (208)	
Other	12.8 (121)	12.0 (84)	15.1 (37)	
Behavioral factors				
Cigarette pack/day (last 6 m), % (n)				0.14
Non-smoker	18.4 (172)	17.8 (124)	19.8 (48)	
< 1 pack per day	57.7 (541)	59.6 (414)	52.5 (127)	
> = 1 pack per day	23.9 (224)	22.6 (157)	27.7 (67)	
Injection drug use, % (n)	**37.1 (351)**	**35.0 (245)**	**43.3 (106)**	**0.02**
Comorbid medical conditions				
BMI, kg/m^2^, median (IQR)	**25.6 (22.5, 29.7)**	**25.9 (22.7, 30.1)**	**24.4 (21.6, 28.6)**	** < 0.01**
HIV positive, % (n)	**44.4 (420)**	**41.2 (289)**	**53.5 (131)**	** < 0.01**
HCV positive, % (n)	**85.3 (807)**	**83.9 (588)**	**89.4 (219)**	**0.04**
Prevalent hypertension, % (n)	46.5 (439)	45.9 (321)	48.2 (118)	0.53
Prevalent diabetes, % (n)	14.5 (80)	14.5 (61)	14.4 (19)	0.97

*p value was tested by Chi-square test in categorical variables, by t tests for normally distributed continuous variable (age), by Wilcoxon tests for skewed continuous variable (BMI).

Significant values are in [bold].

### Level of mtDNA CN with incident CKD and proteinuria

**Figure 2:**
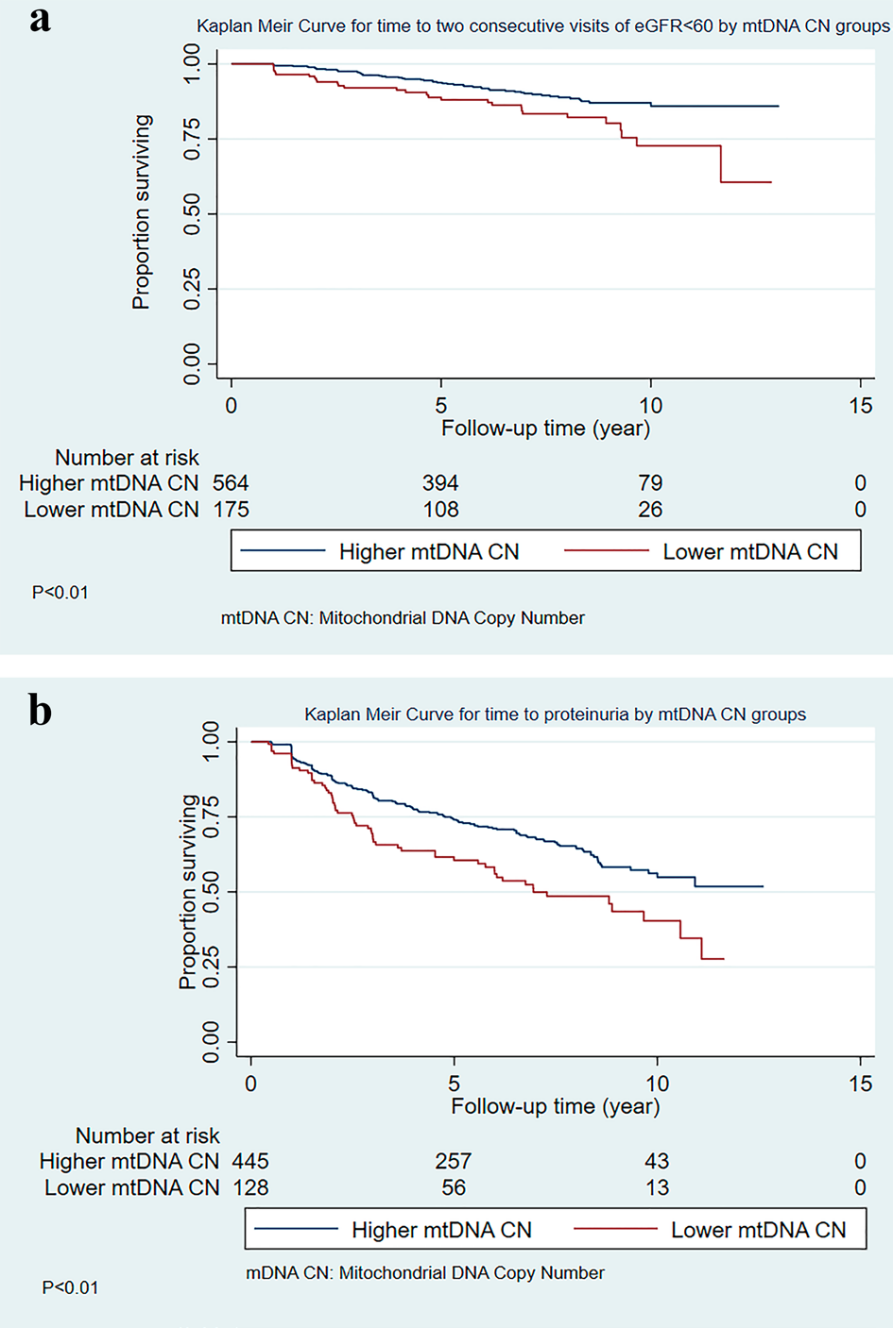
(**a**) Time to incident chronic kidney disease by mitochondrial DNA copy number (mtDNA CN) category in ALIVE. (**b**) Time to incident proteinuria by mitochondrial DNA copy number (mtDNA CN) category in ALIVE.

Among 739 participants without prevalent CKD at baseline, we evaluated time to incident CKD defined by two consecutive visits of eGFR < 60. Participants had a median follow up of 7.42 years (IQR:3.90,8.70) and 82 participants (11%) developed incident CKD by the end of follow-up. Participants with low mtDNA CN had higher likelihood of incident CKD (Log rank test, *P* value < 0.01) (Fig. 2a). Low mtDNA CN was significantly associated with higher hazard of incident CKD in the unadjusted model (HR: 1.79, 95% CI 1.12, 2.85, *P* value = 0.01). After adjustment for covariates, being in the low quartile of mtDNA CN was associated with an adjusted HR of 2.33 (1.42, 3.80) for risk of CKD (*P* value < 0.01, Table 2). The strongest confounding factors in the models was the baseline eGFR while adjusting for other covariates had no effect on the significance and minimal impact on the change in magnitude of the estimate. We also conducted a sensitivity analysis to evaluate the hazard of incident CKD and mtDNA CN using the updated race neutral eGFR formula, and the results were consistent with our primary analysis (Supp. Table 1).

**Table 2:** Risk of chronic kidney disease and proteinuria by mitochondrial DNA copy number (mtDNA CN) groups in ALIVE participants.

Model	Higher Quartiles (1,2,3) of mtDNA CN	Lowest quartile of mtDNA CN Hazard Ratio (95% CI)	P value
Analysis A. Outcome: Incident chronic kidney diseases*			
A^1^	Ref	1.79 (1.12,2.85)	0.01
B^2^	Ref	2.23 (1.39, 3.57)	< 0.01
C^3^	Ref	2.24 (1.40,3.60)	< 0.01
D^4^	Ref	2.17 (1.34,3.50)	< 0.01
E^5^	Ref	2.33 (1.42, 3.80)	< 0.01
Analysis B. Outcome: Incident proteinuria¶			
A^1^	Ref	1.65 (1.22,2.24)	< 0.01
B^2^	Ref	1.67 (1.23,2.27)	< 0.01
C^3^	Ref	1.51 (1.10,2.06)	< 0.01
D^4^	Ref	1.44 (1.05,1.97)	0.02
E^5^	Ref	1.42 (1.04,1.96)	0.03

*Incident chronic kidney disease was defined by first occurrence of two consecutive visits with eGFR < 60.

¶Incident proteinuria was defined by the first occurrence of ratio of urine protein to creatinine > 200.

^1^Crude estimates of the association between mtDNA CN and risk of chronic kidney diseases.

^2^Model adjusted for age, sex, race, baseline eGFR.

^3^Model adjusted for all covariates in Model^2^ and HCV and HIV status.

^4^Model adjusted for all covariates in Model^3^ and cigarette use, injection drug use.

^5^Model adjusted for all covariates in Model^4^ and hypertension and diabetes.

We also evaluated time to first occurrence of ≥ 40% drop in baseline eGFR (incident kidney injury) as part of our sensitivity analysis to explore whether low mtDNA CN was additionally associated with relatively transient kidney function drop (Supp. Table 2, Supp Fig. 2). Low mtDNA CN was also associated with risk for ≥ 40% eGFR decline from baseline (crude HR: 1.54, 95% CI 1.04, 2.27) (Supp. Table 2). After adjustment to all covariates, the association was not significant.

Of 573 individuals without prevalent proteinuria at baseline, with a median of 5.9 (2.5, 8.2) years of follow-up, 209 (36%) developed proteinuria during follow-up. The low mtDNA CN group had higher risk of incident proteinuria compared to the higher mtDNA CN group (Log rank test, *P* < 0.01) (Fig. 2b). Low mtDNA CN was significantly associated with higher hazard of incident proteinuria in the unadjusted model (HR: 1.65, 95% CI 1.22, 2.24, *P* value < 0.01, Table 2). After adjustment, being in the low quartile of mtDNA CN was associated with an adjusted HR of 1.42 (1.04, 1.96) for risk of CKD (*P* value = 0.03, Table 2).

### Change in kidney function over time by level of mtDNA CN

**Figure 3:**
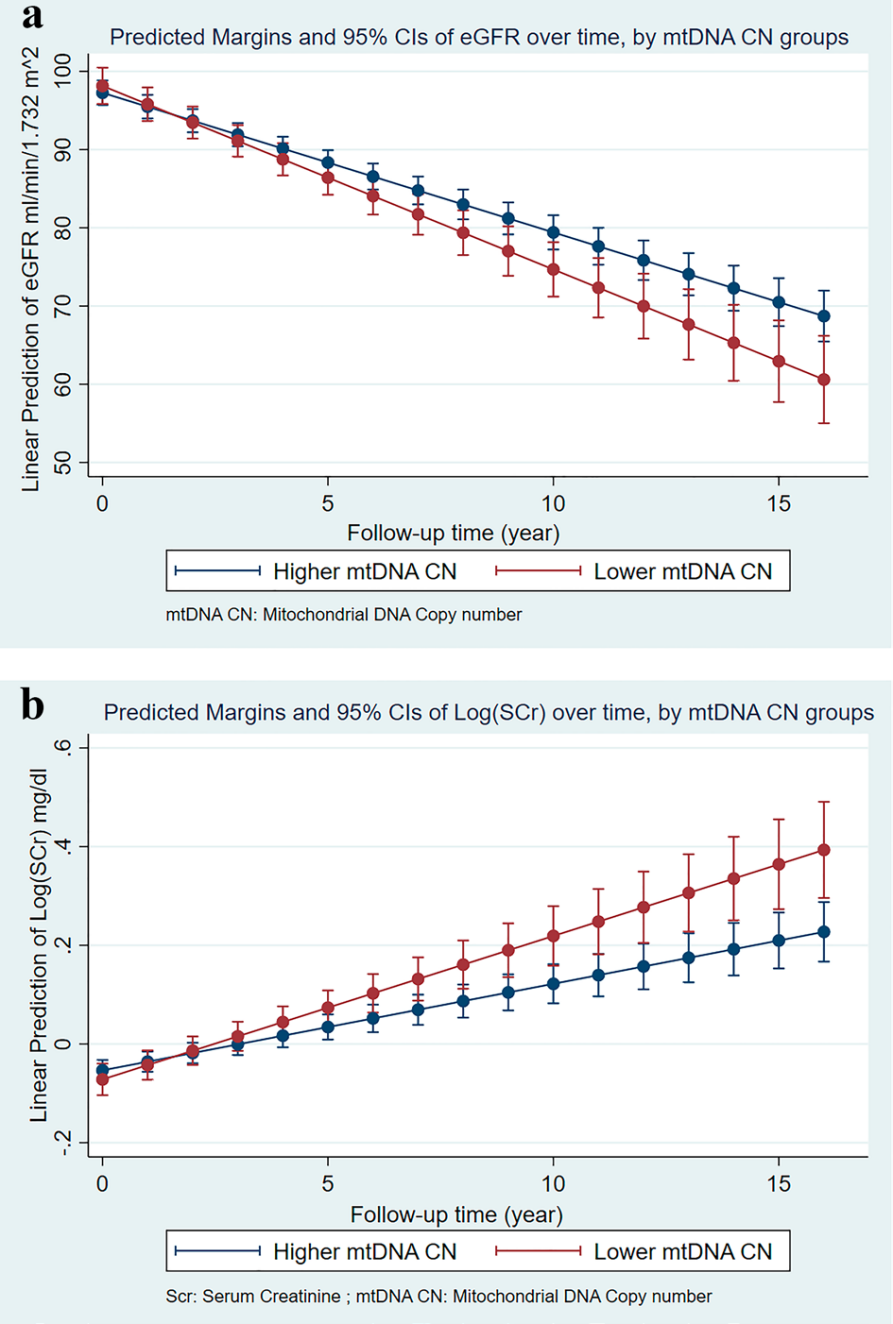
(**a**) Predicted margins and 95% confidence intervals for estimated Glomerular Filtration Rate (eGFR) over time by mitochondrial DNA copy number (mtDNA CN) groups in ALIVE. (**b**) Predicted margins and 95% confidence intervals for estimated Serum Creatinine (Scr) over time by mitochondrial DNA copy number (mtDNA CN) groups in ALIVE.

Overall, eGFR declined in the population at a rate of 1.78 ml/min/1.732 m^2^ each year (*P* value < 0.01, Table 3). Although the mean eGFR was not significantly different between mtDNA CN categories, the interaction of mtDNA CN with time demonstrates that eGFR declined additional 0.56 ml/min/1.732 m^2^ per year faster among participants with low mtDNA CN than those who had higher mtDNA CN (*P* value = 0.01, Fig. 3a). Adjusting for HIV status, HCV status, cigarette use and injection drug use resulted in minimal change in effect estimates.

**Table 3:** Longitudinal association between Mitochondrial DNA Copy number (mtDNA CN) and Kidney function in ALIVE.

Kidney function	Crude estimate			Adjusted estimate	
	Variable	Estimate (95% CI)	*P* value	Estimate^a^ (95% CI)	*P* value
Trajectory of eGFR					
	Low mtDNA CN (Q4)	0.56 (− 1.84, 2.97)	0.65	0.88 (− 1.54, 3.31)	0.48
	Time (year)	− 1.77 (− 1.98, − 1.56)	< 0.01	− 1.78 (− 1.99, − 1.57)	< 0.01
	Low mtDNA CN and time interaction	− 0.53 (− 0.97, − 0.09)	0.02	− 0.56 (− 1.00, − 0.11)	0.01
Trajectory of log transformed serum creatinine					
	Low mtDNA CN (Q4)	− 0.005 (− 0.042, 0.032)	0.79	− 0.018 (− 0.052, 0.015)	0.29
	Time (year)	0.018 (0.014,0.022)	< 0.01	0.018 (0.013, 0.021)	< 0.01
	Low mtDNA CN and time interaction	0.01 (0.002,0.018)	0.02	0.012 (0.004, 0.018)	< 0.01

*Adjusted models for eGFR (ml/min/1.732 m^2^) controlled for baseline age, hypertension, diabetes, cigarette use, injection drug use, HIV status and HCV status; Adjusted models for log (Serum creatinine) (mg/dl) controlled for baseline age, sex, race, hypertension, diabetes, cigarette use, injection drug use, HIV status and HCV status.

*mtDNA CN measurement has been standardized and adjusted for white blood cell counts and platelet counts. Low mtDNA CN indicated the lowest quartile of mtDNA CN within the study participants.

^a^Indicates that the estimate is adjusted.

Serum creatinine increased at 0.018 log mg/dl per year (*P* value < 0.01, Table 3). While the mean serum creatinine was not significantly different, participants with low mtDNA CN had serum creatinine increase at a greater rate with an additional 0.012 log mg/dl per year (*P* value < 0.01) compared to participants with higher mtDNA CN (Fig. 3b). Similarly, baseline age, diabetes and hypertension were significantly associated with trajectories of serum creatinine. A sensitivity analysis was carried out by restricting the linear mixed-effects model to study visits that were within 5 years of the last visit with mtDNA CN measurements. The results were consistent with our primary analysis. (Supp. Table 3).

In all analyses, we further tested whether HIV is an effect modifier of the association between mtDNA CN and incident CKD, incident proteinuria, and trajectories of kidney function decline (eGFR and serum creatinine) by conducting stratified analyses by HIV status and by integrating statistical interaction terms. We did not observe a heterogeneity of effect in models among people with and without HIV (data not shown).

## Discussion

In a large, long-standing cohort of PWID with or at risk of HIV, we demonstrated that low mtDNA CN was associated with greater kidney function decline and greater hazard of developing CKD and proteinuria during long-term follow-up. We observed that the associations were independent of traditional risk factors for CKD and PWID population characteristics. Our results suggest that characteristics of mitochondrial organelles such as mtDNA CN may be associated with creatinine clearance and glomerular filtration. Our data also filled an important gap in the literature for a marginalized high-risk population of PWID and demonstrated consistent findings irrespective of HIV status. Collectively, these results highlighted the importance of studying mitochondrial function and characteristics in kidney diseases.

Low mtDNA CN is a proxy for mitochondrial dysfunction^46^ and peripheral blood derived mtDNA CN can reflect systemic mtDNA dysfunction^39,55^. Our results are consistent with findings from the ARIC study, which found mtDNA CN was associated with incidence of CKD^57^. Compared to the ARIC study, we further provided evidence that level of mtDNA CN is associated with incident proteinuria and longitudinal trajectories of kidney function decline.

Among ALIVE participants, individuals who were actively injecting, HIV-positive, HCV seropositive and had lower BMI had significantly lower level of mtDNA CN compared to their counterparts at baseline. Molecular aspects of illicit drug use, HIV viremia, and HCV co-infection act as inflammatory stimuli that result in increased ROS production via multiple cellular organelles of which, mitochondria are the biggest source. Results from various in vitro studies offer evidence of the effect of mitochondrial dysfunction on kidney function^37,39,40^. Healthy mitochondrial dynamics are marked by a homeostasis of mitochondrial fission and fusion which allows cells to adapt to external stressors to meet ATP needs^81^. Induced imbalance in these processes by either viral infection (i.e. HIV or HCV) and/or injection drug use related stressors could result in ROS-mediated nucleoid clustering by targeting mtDNA stabilization proteins such as TFAM, which would then potentially affect mtDNA replication^48–50^. In turn, reduced mtDNA replication could not only imbalance ROS, but also impact ATP energy reserves via under-activity of ETC complexes^82^. The kidneys are second only to the heart in energy consumption per gram of tissue^83^. Depletion of mtDNA may be detrimental to the vital process of urinary waste removal. Additionally, mtDNA dysfunction could affect tissue injury repair processes involving immune cells^27,29,30^^(p4)^. As kidney disease is one of the leading causes of hospitalization and death among PWIDs^84–86^, our findings warrant continued investigation of mtDNA CN and substance use (i.e. opioids, cocaine) to improve long-term quality of life in this vulnerable population.

Sequential adjustment of cox regression models for incident CKD and proteinuria allowed glimpses into relative strength of confounding and directions of adjustment variables. In our analysis, baseline eGFR (CKD Model B, Table 2) most strongly negatively confounded the mtDNA and CKD association. Adjusting for baseline eGFR accounts for the baseline kidney function, which is important to factor in before comparing time to CKD. In our population, baseline eGFR additionally captures risk history (eg: kidney burden due to history of illicit drug use, ART, and sociodemographic/systemic factors etc.) of each participant, thereby reducing impact of unmeasured confounders and strengthening the association. In our sensitivity analyses of a ≥ 40% eGFR decline outcome, behavioral factors of cigarette use and injection drug use created maximum changes in the effect estimate upon adjustment. It is unclear to what extent the interaction between risk behaviors, such as cigarette use and injection drug use, may impact kidney injury over time. Future investigation may want to focus on the independent and aggregated effect of these risk behaviors in the association between mitochondrial function and kidney function outcomes.Conversely, the association of mtDNA CN with proteinuria was attenuated post adjustment, most strongly by HIV/HCV infection status (Proteinuria Model C, Table 2). Attenuation implies positive confounding by these variables, i.e., those with HIV or HCV infection are likely to have low mtDNA CN as well proteinuria due to infection related characteristics and risk behaviors among PWIDs.

Our study has certain limitations. First, although we controlled for potential confounders of CKD, unmeasured confounders may still exist. Although we did not focus on ART exposures, we controlled for HIV effects in the models and evaluated models stratified by HIV. Future analyses on the impact of ART and specific regimen on mitochondrial DNA copy number will be warranted. Second, since mtDNA CN was measured only at two timepoints 5 years apart and carried forward between visits, we assumed the mtDNA CN and the impact on kidney disease remained constant over time. We did not evaluate impact of medications that may lead to kidney injury or treatment of kidney diseases, the use of dialysis or systemic and social factors of socio-economic status, healthcare access in our analyses. Our future studies will focus on the interplay of medication and mitochondrial function on kidney diseases.

Albeit with the limitations, our study is one of the most comprehensive evaluations of the longitudinal association between mtDNA CN and kidney diseases to date, including CKD, proteinuria, and longitudinal trajectories of kidney function. Leveraging the ALIVE data and infrastructure, we were able to assess longitudinal changes of kidney function in a large cohort setting with the ability to control for refined time-varying bio-behavioral characteristics of the participants, such as existing comorbidities (hypertension, diabetes, BMI, HIV, HCV), behavioral characteristics (injection drug use, smoking intensity) and demographics (age, sex race) Our categorization of hypertensive and diabetic participants accounted for medication use in addition to clinical cut-offs. Additionally, the mtDNA CN measurements were adjusted for cell composition and random effects of plating and pipetting, which may provide more robust estimates of the systematic mitochondrial function compared to some of the previous studies. Finally, our data fills an important gap in the literatures among an otherwise hard to reach population of PWIDs with and without HIV. This vulnerable population has a heavy burden of terminal kidney diseases and yet has not been well studied in previous research.

Our results demonstrate that low level of mtDNA CN in peripheral blood is independently associated with risk of CKD and proteinuria, as well as faster kidney function decline among PWID. CKD characterization among PWID is unique, attributable to additional risk behaviors and internal mechanisms. By accounting for some of these factors in our analyses, we add to the PWID literature a comprehensive picture of the mtDNA CN and CKD association. Keeping in mind the kidney burden in this population, our findings provide insight on potential mechanisms of CKD development and potentially enrich current understanding of CKD biomarkers, both of which have implications in CKD screening, monitoring, and risk stratification tools. More research is warranted to evaluate the interplay of mitochondrial characteristics and function with substance abuse and their roles in the etiology of kidney diseases.

### Supplementary Information

Supplementary Information.

## Data Availability

Data will be available upon request, for more information contact Dr Jing Sun (jsun54@jhmi.edu).
