# Supplementary material for: Mitochondrial DNA copy number is associated with incident chronic kidney disease and proteinuria in the AIDS linked to the intravenous experience cohort
Source: Sci Rep. 2023 Oct 27;13:18406. doi: 10.1038/s41598-023-45404-9 (PMC10611749; doi:10.1038/s41598-023-45404-9)
Supplement: Supplementary file 1 — Supplementary Information. [file 41598_2023_45404_MOESM1_ESM.docx]

Supplementary Figure 1. The association between mitochondrial DNA copy number by quartiles and trajectories of kidney function in ALIVE participants.


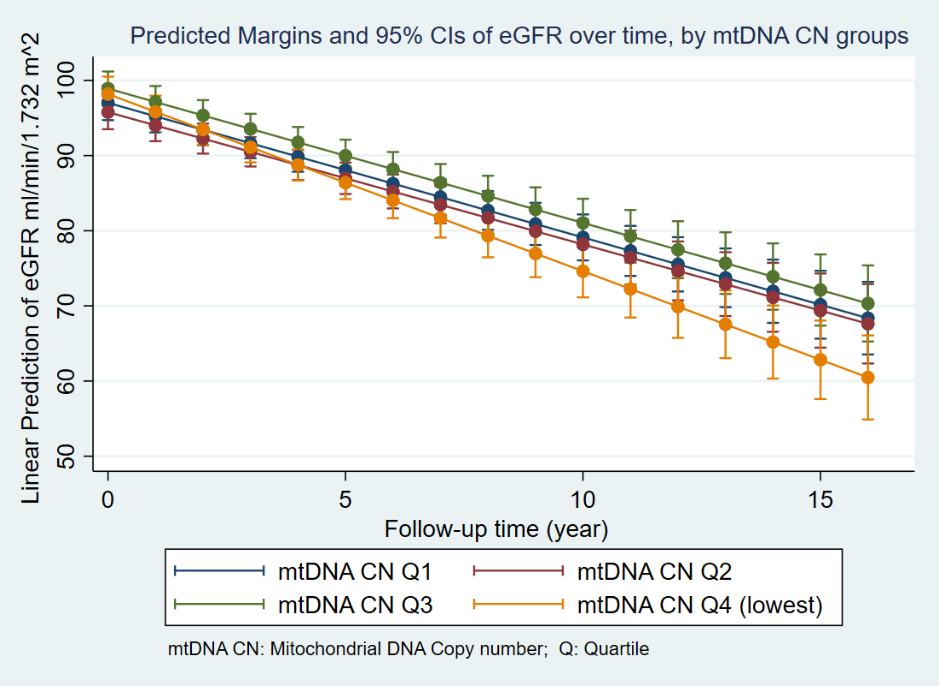

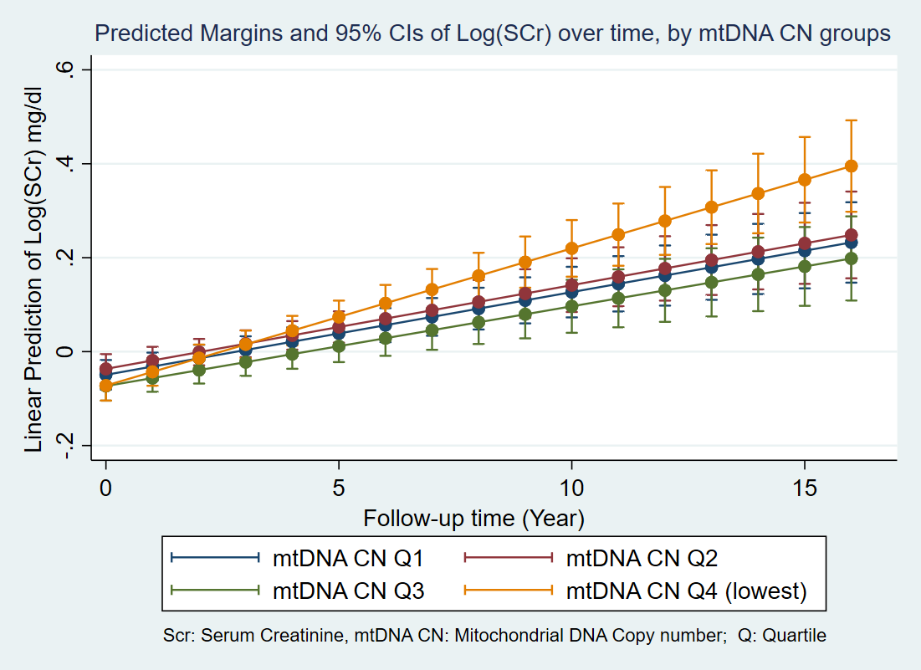


Supplementary 1b. Trajectory of serum creatinine

Supplementary 1a. Trajectory of eGFR

Supplementary Figure 2. Time to kidney injury* by mitochondrial DNA copy number groups among ALIVE participants.


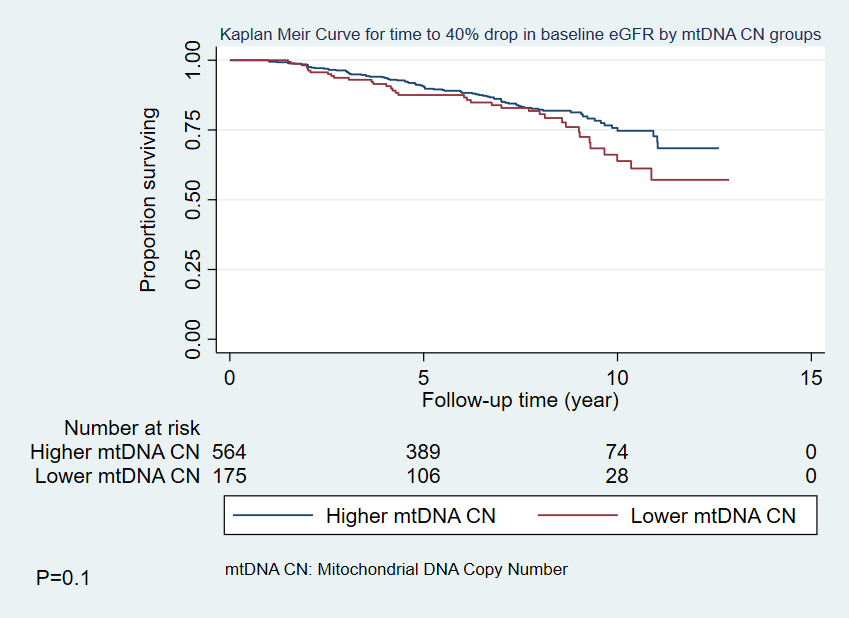


*Kidney injury is defined by first occurrence of ≥40% drop in baseline eGFR

Supplementary table 1: Sensitivity analysis: Risk of incident chronic kidney diseases (defined by the updated race neutral eGFR formula) among ALIVE participants by mitochondrial DNA copy number groups

| eGFR: First occurrence of two consecutive GFR<60 | | | |
| --- | --- | --- | --- |
| Model | Quartiles 1,2,3 | Low mtDNA CN (Q4) HR (95% CI) | P value |
| A^1^ | Ref | 1.53 (1.01, 2.30) | 0.040 |
| B^2^ | Ref | 1.75 (1.15, 2.64) | 0.008 |
| C^3^ | Ref | 1.71 (1.12, 2.60) | 0.012 |
| D^4^ | Ref | 1.63 (1.07, 2.48) | 0.022 |
| E^5^ | Ref | 1.68 (1.10, 2.57) | 0.020 |

eGFR: ^1^Unadjusted; ^2^Adjusted for age, sex, race, baseline eGFR; ^3^Added HCV and HIV status; ^4^Added cigarette use, injection drug use; ^5^Added Hypertension and Diabetes.

Supplementary Table 2: Risk of kidney injury by mitochondrial DNA copy number groups among ALIVE participants

| Kidney injury: at least a 40% drop in baseline GFR | | | |
| --- | --- | --- | --- |
| Model | Quartiles 1,2,3 | Low mtDNA CN (Q4) HR (95% CI) | P value |
| A^1^ | Ref | 1.54 (1.04,2.27) | 0.030 |
| B^2^ | Ref | 1.55 (1.04,2.28) | 0.029 |
| C^3^ | Ref | 1.48 (0.99,2.19) | 0.051 |
| D^4^ | Ref | 1.39 (0.93,2.08) | 0.103 |
| E^5^ | Ref | 1.48 (0.98,2.22) | 0.060 |

eGFR: ^1^Unadjusted; ^2^Adjusted for age, sex, race, baseline eGFR; ^3^Added HCV and HIV status; ^4^Added cigarette use, injection drug use; ^5^Added Hypertension and Diabetes.

Supplementary Table 3. Longitudinal association between Mitochondrial DNA Copy number and Kidney function in ALIVE restricting study visits to renal measurements within 5 years of the last mitochondrial DNA copy number measurement.

| Kidney Function | Crude Estimate | | | Adjusted Estimate^a^ | | |
| --- | --- | --- | --- | --- | --- | --- |
|  | Variable | Estimate (95% CI) | P value | Variable | Estimate (95% CI) | P value |
| eGFR |  |  |  |  |  |  |
|  | Low mtDNA CN (Q4) | 0.62 (-2.13, 3.37) | 0.66 | Low mtDNA CN (Q4) | 0.83 (-1.92, 3.58) | 0.56 |
|  | Time (year) | -1.83 (-2.13, -1.53) | <0.01 | Time (year) | -1.83 (-2.12, -1.53) | <0.01 |
|  | Low mtDNA CN and time interaction | -0.52 (-1.11, 0.08) | 0.09 | Low mtDNA CN and time interaction | -0.55 (-1.14, 0.03) | 0.06 |
| Log (Serum Creatinine) |  |  |  |  |  |  |
|  | Low mtDNA CN (Q4) | -0.003 (-.04, 0.04) | 0.88 | Low mtDNA CN (Q4) | -0.02 (-0.06, 0.02) | 0.28 |
|  | Time (year) | 0.02 (0.013,0.025) | <0.01 | Time (year) | 0.017 (0.012, 0.02) | <0.01 |
|  | Low mtDNA CN and time interaction | 0.008 (-0.002,0.018) | 0.13 | Low mtDNA CN and time interaction | 0.011 (.003, 0.019) | 0.01 |

*Adjusted models for eGFR (ml/min/1.732 m^2^) controlled for baseline age, hypertension, diabetes, cigarette use, injection drug use, HIV status and HCV status; Adjusted models for Serum creatinine (mg/dl) controlled for baseline age, sex, race, hypertension, diabetes, cigarette use, injection drug use, HIV status and HCV status

*mtDNA CN measurement has been standardized and adjusted for white blood cell counts and platelet counts.

*Models are restricted to renal observations within 5 years of last mtDNA measurements

Supplementary Table 4. Longitudinal association between Mitochondrial DNA Copy number (mtDNA CN) and Kidney function in ALIVE, with mtDNA CN modeled as quartiles.

| eGFR | Crude Estimate | | |
| --- | --- | --- | --- |
|  | Quartiles (Q) | Estimate (95% CI) | P value |
| Low mtDNA CN and time interaction | Q1 | Ref |  |
|  | Q2 | -0.03 (-0.53,0.48) | 0.92 |
|  | Q3 | 0.0067 (-0.49,0.50) | 0.97 |
|  | Q4 | -0.55 (-1.07,-0.02) | 0.039 |

Supplementary Table 5: Abbreviation index

| ***Abbreviation*** | ***Definition*** |
| --- | --- |
| CKD | Chronic Kidney Disease |
| HIV | Human Immunodeficiency Virus |
| PWH | People With HIV |
| ART | Antiretroviral Therapy |
| HCV | Hepatitis C Virus |
| PWID | Person Who Injects Drugs |
| ROS | Reactive Oxygen Species |
| ATP | Adenosine Tri Phosphate |
| OXPHOS | Oxidative Phosphorylation |
| MtDNA | Mitochondrial DNA |
| CN | Copy Number |
| ARIC | Atherosclerosis Risk in Communities |
| ESRD | End-Stage Renal Disease |
| eGFR | Estimated Glomerular Filtration Rate |
| ALIVE | AIDS Linked to the Intravenous Experience |
| STROBE | Strengthening the Reporting of Observational Studies in Epidemiology |
| UPCR | Urine Protein to Creatinine Ratio |
| qPCR | Quantitative Polymerase Chain Reaction |
| WBC | White Blood Cell |
| IQR | Inter Quartile Range |
| BMI | Body Mass Index |
